# Development and validation of GMI signature based random survival forest prognosis model to predict clinical outcome in acute myeloid leukemia

**DOI:** 10.1186/s12920-019-0540-5

**Published:** 2019-06-26

**Authors:** Mingguang Shi, Guofu Xu

**Affiliations:** School of Electric Engineering and Automation, Hefei University of Technology, Hefei, 230009 Anhui China

**Keywords:** Acute myeloid leukemia, A GMI signature, The functional miRNA-mRNA regulatory module, Random survival forest

## Abstract

**Background:**

Acute myeloid leukemia (AML) is a disease with marked molecular heterogeneity and a high early death rate. Our aim was to investigate an integrated Gene expression, Mirna and miRNA-mRNA Interactions (GMI) signature for improving risk stratification of AML.

**Methods:**

We identified differentially expressed genes by pooling a large number of 861 human AML patients and 75 normal cases. We then used miRWalk to identify the functional miRNA-mRNA regulatory module. The GMI signature based random survival forest (RSF) prognosis model was developed from training data set and evaluated in independent patient cohorts from The Cancer Genome Atlas (TCGA) dataset (*N* = 147). Univariate and multivariate Cox proportional hazards regression analyses were applied to evaluate the prognostic value of GMI signature.

**Results:**

We identified 139 differentially expressed genes between normal and abnormal AML samples. We discovered the functional miRNA-mRNA regulatory module which participate in the network of cancer progression. We named 23 differentially expressed genes and 16 validated target miRNAs as the GMI signature. The RSF model-based scores separated independent patient cohorts into two groups with significantly different overall survival (C-index = 0.59, hazard ratio [HR], 2.12; 95% confidence interval [CI], 1.11–4.03; *p* = 0.019). Similar results were obtained with reversed training and testing datasets (C-index = 0.58, hazard ratio [HR], 2.08; 95% confidence interval [CI], 1.02–4.24; *p* = 0.038). The GMI signature score contributed more information about recurrence than standard clinical covariates.

**Conclusion:**

The GMI signature based RSF prognosis model not only reflects regulatory relationships from identified miRNA-mRNA module but also informs patient prognosis. While in the TCGA data set the GMI signature score contributed additional information about recurrence in comparison to standard clinical covariates, further studies are needed to determine its clinical significance.

**Electronic supplementary material:**

The online version of this article (10.1186/s12920-019-0540-5) contains supplementary material, which is available to authorized users.

## Background

Acute myeloid leukemia (AML) is a malignant disease of the bone marrow and typically represents functionally and phenotypically various cells in the same patient. Gene mutations identified distinct cytogenetically defined subsets of AML and unraveled the heterogeneous disorder in terms of genetic basis. Karyotype [1], mutations in the transcription factor CCAAT/enhancer-binding protein alpha (*CEBPA*) [2, 3], internal tandem duplications of the fms-related tyrosine kinase 3 (*FLT3-ITD*) [4], recurrent lesions in the nucleophosmin gene (*NPM1*) [5], *GATA* binding protein 2(*GATA2*) mutations [6], and Wilms tumor 1 (*WT1*) mutations [7] are related with patient relapse, prognosis and survival outcome. Although these guidelines for clinical treatment have improved the prognosis, AML is curable in about 35% of patients under 60 years old and 10% over 60 years old [8]. Hence, it is crucial to develop a reliable method for identifying new biomarkers and developing prognosis model to guide individual treatment of patients.

Several methods have been developed for the analysis of multiple molecular data to identify cancer-driven signatures and predict clinical outcome. Previous studies have demonstrated that microRNA signatures were identified to be associated with cytogenetics, prognosis and therapeutic targets in AML [9–11] and microRNA expression-based model could predict event-free survival in AML [12, 13]. Moreover, distinct molecular subgroups that reflect discrete paths in the evolution of AML was identified to inform disease classification and prognostic stratification [14]. High-throughput proteomics data, such as Reverse Phase Proteomic Arrays (RPPA), was utilized to develop the prognosis model and bridge the gap between the underlying genetic alterations and functional cellular changes [15]. With the advances in next-generation sequencing (NGS) studies, integration of multiple molecular data and genomic knowledge improved the understanding of molecular pathogenesis and underlying biology in cancer [16]. Integrative data analysis methods led to the identification of novel microRNA-target gene interactions of potential relevance [17] and the discovery of NPM1 mutation-modulated miRNA-mRNA regulation pairs [18] for AML treatment. However, the sample size is still relatively small and the prognosis model has not been mentioned for patient stratification.

To address these questions, we collected 25 publicly available gene expression data sets containing 861 human AML patients and 75 normal cases. By pooling such a large amount of data, we aimed to identify differentially expressed genes for describing different gene expression patterns between normal samples and AML samples. Furthermore, we wanted to discover the functional regulatory networks for identifying potential regulations between mRNA and miRNA in biological processes. Based on the observation that miRNA-mRNA interactions were biologically relevant, we hypothesized that a functional miRNA-mRNA regulatory module could better represent underlying biological characteristics and may lead to a more powerful prognosis model. Moreover, we tried to develop Random survival forest (RSF) model using The Cancer Genome Atlas (TCGA) dataset based on prognostic miRNA/mRNA signatures. The results demonstrated that the model-based patient stratification provided useful predictive information for AML patient subgroups.

## Methods

### Study design

**Fig. 1:**
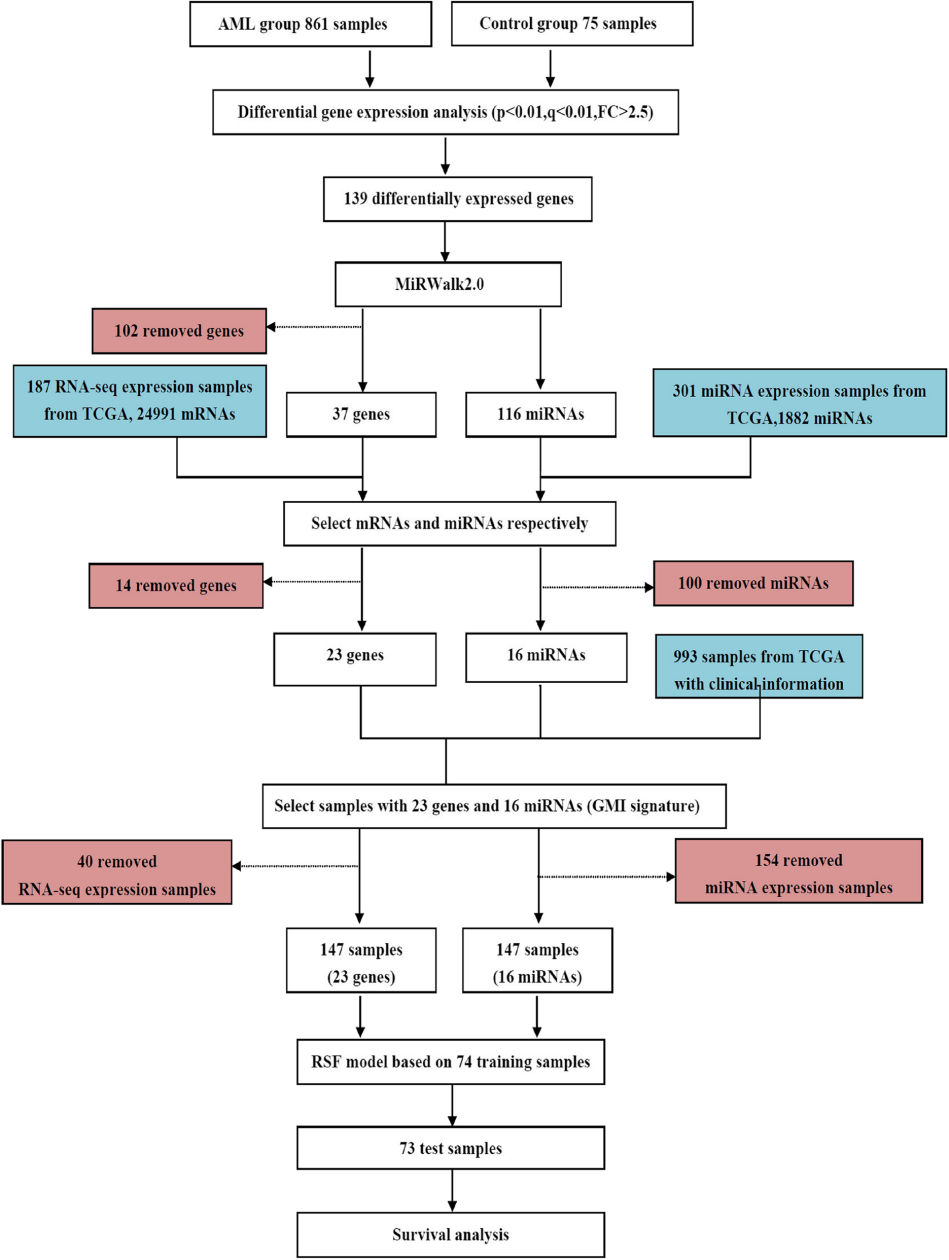
Outline of the study design. Differentially expressed genes were identified with differential expression analysis. MiRWalk was used to reconstruct the functional miRNA-mRNA regulatory module. RSF method was then used with GMI signature to develop prognosis model in training cohort. The developed prognosis model was evaluated on the independent dataset. The gained RSF-based score was applied for survival analysis and patient stratification. Specifically, we removed 14 genes which were not found in 187 RNA-seq expression dataset and 100 miRNAs which were not found in 301 miRNA expression dataset. Samples from TCGA with no expression of the signatures genes/miRNAs were filtered out. One hundred forty-seven AML samples were selected based on the present expression values of 23 gene expression signatures from 187 RNA-seq expression dataset and 16 miRNAs from 301 miRNA expression dataset

Figure 1 illustrated the overview of the overall study design. Microarray gene expression data on AML were collected and processed. Differential gene expression analysis was performed to identify differentially expressed genes. MiRWalk was then used to discover miRNAs that target the prioritized genes and further identify the functional miRNA-mRNA regulatory module. Specifically, the list of differentially expressed 23 genes and 16 target-validated miRNAs were named as the GMI signature because it integrated information from Gene expression, Mirna and miRNA-mRNA Interactions. Random survival forest (RSF) method were used with GMI signature to develop prognosis model in training cohort and then evaluated on the test dataset. We used RNA-seq and miRNA expression dataset on AML cases from TCGA as training and validation cohort. The gained RSF-based score was applied for patient stratification and survival analysis.

### Sample collection of AML datasets

We searched PubMed (https://www.ncbi.nlm.nih.gov/pubmed) with the terms “acute myeloid leukemia”, “gene expression”, “prognosis” and “signature” for published articles. This search retrieved the relevant Gene Expression Omnibus (GEO) database for further analysis. Raw microarray data were obtained from publicly available AML gene expression datasets in NCBI GEO. We collected AML samples of 25 gene expression datasets with Affymetrix Human Genome U133 plus 2.0 microarrays. The normalization was conducted on all samples in a single set using Robust MultiChip Analysis (RMA) algorithm [19] and Quantile Normalization and thus ensured the independence of processed datasets. Signal intensities were log2 transformed and probe set values summarized using Median Polish Summarization Method [20]. Probe set identifiers (IDs) were mapped to gene symbols based on the mapping from the GEO database. To resolve redundancies, multiple probes were mapped to unique Entrez Gene IDs by their median expression level. To make expression level comparable across genes, expression values for each gene were standardized using a Z-score transformation. An experimental group including 861 AML samples and a control group containing 75 normal bone marrow samples were used for the prioritization of gene expression signatures. The information of the source datasets of all AML samples is shown in the Table 1.

**Table 1:** AML gene expression datasets used to prioritize the gene expression signatures

Data Set	Country	Control group	Experimental Group	Author	Title	Journal	Reference
GSE14924	United Kingdom	21	20	(Le et al. 2009)	Peripheral blood T cells in acute myeloid leukemia (AML) patients at diagnosis have abnormal phenotype and genotype and form defective immune synapses with AML blasts	Blood	[21]
GSE68172	Germany	5	72	(Schneider et al. 2015)	Leukemic progenitor cells are susceptible to targeting by stimulated cytotoxic T cells against immunogenic leukemia-associated antigens	International Journal of Cancer	[22]
GSE84881	Germany	4	19	(Ek et al. 2016)	Molecular alterations in bone marrow mesenchymal stromal cells derived from acute myeloid leukemia patients	Leukemia	[23]
GSE14858	Italy	20	20	(Bresolin et al. 2010)	Gene expression-based classification as an independent predictor of clinical outcome in juvenile myelomonocytic leukemia	Journal of Clinical Oncology	[24]
GSE12662	USA	15	91	(Payton et al. 2009)	High throughput digital quantification of mRNA abundance in primary human acute myeloid leukemia samples	Journal of Clinical Investigation	[25]
GSE10746	USA	3	8	(Mougeot et al. 2011)	Microarray analyses of oral punch biopsies from acute myeloid leukemia (AML) patients treated with chemotherapy	Oral Surgery Oral Medicine Oral Pathology Oral Radiology & Endodontology	[26]
GSE17054	USA	4	9	(Majeti et al. 2009)	Dysregulated gene expression networks in human acute myelogenous leukemia stem cells	Proceedings of the National Academy of Sciences of the United States of America	[27]
GSE8023	USA	3	9	(Krejci et al. 2008)	p53 signaling in response to increased DNA damage sensitizes AML1-ETO cells to stress-induced death	Blood	[28]
GSE17061	Netherlands	0	35	(Silva et al. 2009)	Gene expression profiling of minimally differentiated acute myeloid leukemia: M0 is a distinct entity subdivided by RUNX1 mutation status	Blood	[29]
GSE70124	Germany	0	46	(Papaemmanuil et al. 2016)	Genomic Classification and Prognosis in Acute Myeloid Leukemia	New England Journal of Medicine	[14]
GSE10258	Austria	0	15	(Zatkova et al. 2009)	AML/MDS with 11q/MLL amplification show characteristic gene expression signature and interplay of DNA copy number changes	Genes Chromosomes & Cancer	[30]
GSE35159	Japan	0	12	(Saito et al. 2011)	CD52 as a molecular target for immunotherapy to treat acute myeloid leukemia with high EVI1 expression	Leukemia	[31]
GSE50928	France	0	13	(Khaznadar et al. 2015)	Defective NK Cells in Acute Myeloid Leukemia Patients at Diagnosis Are Associated with Blast Transcriptional Signatures of Immune Evasion	Journal of Immunology	[32]
GSE34885	France	0	14	(Khaznadar et al. 2015)	Defective NK Cells in Acute Myeloid Leukemia Patients at Diagnosis Are Associated with Blast Transcriptional Signatures of Immune Evasion	Journal of Immunology	[32]
GSE52891	Netherlands	0	23	(Bachas et al. 2015)	Gene Expression Profiles Associated with Pediatric Relapsed AML	Plos One	[33]
GSE22056	Netherlands	0	98	(de Jonge et al. 2010)	High VEGFC expression is associated with unique gene expression profiles and predicts adverse prognosis in pediatric and adult acute myeloid leukemia	Blood	[34]
GSE59808	USA	0	32	(Guo et al. 2014)	PIM inhibitors target CD25-positive AML cells through concomitant suppression of STAT5 activation and degradation of MYC oncogene	Blood	[35]
GSE12326	China	0	10	(Cheung et al. 2009)	A comparative study of bone marrow and peripheral blood CD34+ myeloblasts in acute myeloid leukaemia	British Journal of Haematology	[36]
GSE44857	United Kingdom	0	18	(Leonard et al. 2014)	Sequential Treatment with Cytarabine and Decitabine Has an Increased Anti-Leukemia Effect Compared to Cytarabine Alone in Xenograft Models of Childhood Acute Myeloid Leukemia	Plos One	[37]
GSE30903	Italy	0	24	(Salvestrini et al. 2012)	Purinergic signaling inhibits human acute myeloblastic leukemia cell proliferation, migration, and engraftment in immunodeficient mice	Blood	[38]
GSE22845	Netherlands	0	154	(Taskesen et al. 2011)	Prognostic impact, concurrent genetic mutations, and gene expression features of AML with CEBPA mutations in a cohort of 1182 cytogenetically normal AML patients: further evidence for CEBPA double mutant AML as a distinctive disease entity	Blood	[39]
GSE18018	USA	0	19	(Falini et al. 2010)	Multilineage dysplasia has no impact on biologic, clinicopathologic, and prognostic features of AML with mutated nucleophosmin (NPM1)	Blood	[40]
GSE21261	USA	0	79	(Miesner et al. 2010)	Multilineage dysplasia (MLD) in acute myeloid leukemia (AML) correlates with MDS-related cytogenetic abnormalities and a prior history of MDS or MDS/MPN but has no independent prognostic relevance: a comparison of 408 cases classified as “AML not otherwise specified” (AML-NOS) or “AML with myelodysplasia-related changes” (AML-MRC)	Blood	[41]
GSE56237	Denmark	0	10	(Mora-Jensen et al. 2015)	Cellular origin of prognostic chromosomal aberrations in AML patients	Leukemia	[42]
GSE30442	USA	0	11	(Grossmann et al. 2011)	Whole-exome sequencing identifies somatic mutations of BCOR in acute myeloid leukemia with normal karyotype	Blood	[43]

### TCGA samples for training and validation cohorts

Transcriptomic data from the AML cohort were downloaded from the TCGA site (https://portal.gdc.cancer.gov/). We collected the mRNA sample data (*N* = 187, 24,991 genes), the miRNA sample data (*N* = 301, 1882 miRNA) and the clinical sample data (*N* = 993) of AML from TCGA database. Log transformation (base 2) was used to re-scale mRNA and miRNA expression, followed by a Z-score transformation. Transcriptomic data of GMI signature were obtained and processed from TCGA. Specifically, 147 AML samples were selected based on the present expression values of 23 gene expression signatures from 187 RNA-seq expression dataset and 16 miRNAs from 301 miRNA expression dataset.

Data collection and process was shown in Fig. 1. We split the tissue samples into TCGA Part One (TCGAPO, *N* = 74) and TCGA Part Two (TCGAPT, *N* = 73) cohorts for further analysis. TCGAPO cohort includes 41 samples (alive) and 33 samples (dead) for overall survival (OS) of patients. At the same time, TCGAPT cohort contains 33 samples (alive) and 40 samples (dead) for OS of cancer patients. Each dataset was used as a training-set in turn and developed models were evaluated against the other dataset. Table 2 illustrated the clinical characteristics of TCGA AML cohort.

**Table 2:** The clinical characteristics of AML patients from TCGA. CR, complete remission; MLL, mixed lineage leukemia

	TCGAPO (*N* = 74)	TCGAPT (*N* = 73)
Age at Diagnosis (year)		
median (range)	10.58 (0.40–22.55)	9.04 (0.38–19.12)
Gender		
female (n%)	34 (45.95%)	40 (54.79%)
Race		
white (n%)	59 (79.73%)	55 (75.34%)
First event		
relapse (n%)	51 (68.92%)	54 (73.97%)
CR status		
CR (n%)	69 (93.24%)	61 (83.56%)
Primary Cytogenetic		
MLL (n%)	10 (13.51%)	18 (24.66%)
Normal (n%)	18 (24.32%)	11 (15.07%)
Other (n%)	14 (18.92%)	20 (27.40%)
t(8;21)	7 (9.46%)	11 (15.07%)
inv.(16)	16 (21.62%)	12 (16.44%)
Cytogenetic Site of Relapse/Induction Failure		
Yes (n%)	4 (5.41%)	13 (17.81%)
No (n%)	48 (64.86%)	43 (58.90%)
Not done (n%)	22 (29.73%)	17 (23.29%)
FAB Category		
M0,M1,M2,M3,M4,M5,M6,M7,NOS	2 (2.70%),7 (9.46%),19 (25.68%),0 (0.00%),22 (29.73%),15 (20.27%),1 (1.35%),2 (2.70%),5 (6.76%)	2 (2.74%),8 (10.96%),15 (20.55%),0 (0.00%),20 (27.40%),12 (16.44%),1 (1.37%),5 (6.85%),3 (4.11%)
FLT3/ITD		
Positive (n%)	8 (10.81%)	5 (6.85%)
Negative (n%)	66 (89.19%)	68 (93.15%)
WBC at Diagnosis		
median (range)	53.5 (2.1–302)	34.9 (1.3–519)
NPM mutation		
Yes (n%)	4 (5.41%)	2 (2.74%)
No (n%)	66 (89.19%)	70 (95.89%)
CEBPA mutation		
Yes (n%)	4 (5.41%)	5 (6.85%)
No (n%)	69 (93.24%)	67 (91.78%)
WT1 mutation		
Yes (n%)	4 (5.41%)	6 (8.22%)
No (n%)	67 (90.54%)	66 (90.41%)
Protocol		
CCG-2961(n%), AAML03P1(n%), AAML0531(n%)	18 (24.3%),38 (51.4%),18 (24.3%)	0 (0%),0 (0%),73 (100%)

### Differential gene expression analysis

To identify differentially expressed genes, differential expression analysis was used to assess differences in gene expression between an experimental group and a control group assessed by two-tailed Student’s *t*-tests and corrected by Benjamini-Hochberg [44]. The false discovery rate (FDR) of multiple testing was controlled using the Benjamini and Hochberg method. Significantly differentially expressed genes were selected with the FDR-adjusted *p*-values < 0.01. Adjusted statistical significance was then set at q-values < 0.01 with FDR correction for multiple testing where relevant. Fold Change value of 2.5 was further used as a cut-off to identify up- and down-regulated genes.

### MiRWalk identify miRNA-mRNA interactions

To identify miRNA-mRNA interactions, we used the significantly differentially expressed genes (mRNAs) as seeds and identified the target-validated miRNAs from the miRWalk2.0 database (http://mirwalk.umm.uni-heidelberg.de/) [45]. To investigate the biological relevance of the identified interactions, we used an advanced search options including *miRDB* and *TargetScan* for miRNAs target selection. The functionally correlated miRNA-mRNA regulatory module was then identified and constructed for further analysis.

### Survival analysis for GMI signature

The LinkedOmics database (http://www.linkedomics.org) contains multiple molecular data and clinical data for different cancer types from the TCGA project, which systematically interpret and explore the complex relationships between the vast amount of clinical and molecular attributes [46]. In addition, Gene Expression Profiling Interactive Analysis (GEPIA) (http://gepia.cancer-pku.cn/) was used for efficiently analyzing the RNA sequencing expression data from the TCGA data [47]. In this analysis, we utilized these two analytical tools to perform validation of AML specific expression and prognosis for the GMI signature.

### Statistical analysis

We used random survival forest (RSF) method for developing a prognosis model [48]. An R implementation of the *rfsrc* available in the randomForestSRC package was used for model development. RSF had two parameters *ntree* and *mtry*, where *ntree* represented the number of trees in the forest and *mtry* was the number of randomly selected variables for splitting at each node. We used a grid-search on *ntree* and *mtry* using 5-fold cross-validation. All the pairs of (*ntree*, *mtry*) are formed and the one with the best C-index value is identified as the optimized parameters. The C-index represents a probability of the concordance between predicted and observed survival, which is a typical metric for quantifying the predictive ability of a survival model. The developed RSF prognosis model based on the optimal parameters was then evaluated on the independent dataset where the RSF-based score was derived for each sample.

The calculated C-index values evaluated the association between the RSF-based score and real prognosis of the patients. Standard Kaplan–Meier survival curves were generated for different risk patient groups on the basis of the RSF-based scores. The median score was used to stratify patients into high-risk and low-risk score groups, and the log-rank test was utilized to assess the survival difference between two different risk groups. The statistical test was two-sided and the estimated *p* value less than 0.05 was considered statistically significant.

## Results

### The prioritized gene expression signatures

In the microarray analysis, 139 genes were found to be differentially expressed between 75 normal bone marrow samples and 861 AML samples (Additional file 1: Table S1). All the 139 genes suggested higher expression levels with statistical significance in AML cases than in controls. We illustrated the volcano plot by analyzing genes with differential expressions between 861 AML samples and 75 normal cases for mRNA microarrays (Additional file 2: Figure S1). It showed the significant interactions with −log 10(*p*-value) as a function of the log2 fold-change in the gene expression of AML.

### The functional miRNA-mRNA regulatory module

MiRWalk2.0 is a publicly available comprehensive archive, providing an array of experimentally verified and predicted miRNA-mRNA interaction pairs. It has been proved that miRNA-mRNA interactions play critical roles in diverse biological processes and pathologies [49].

**Fig. 2:**
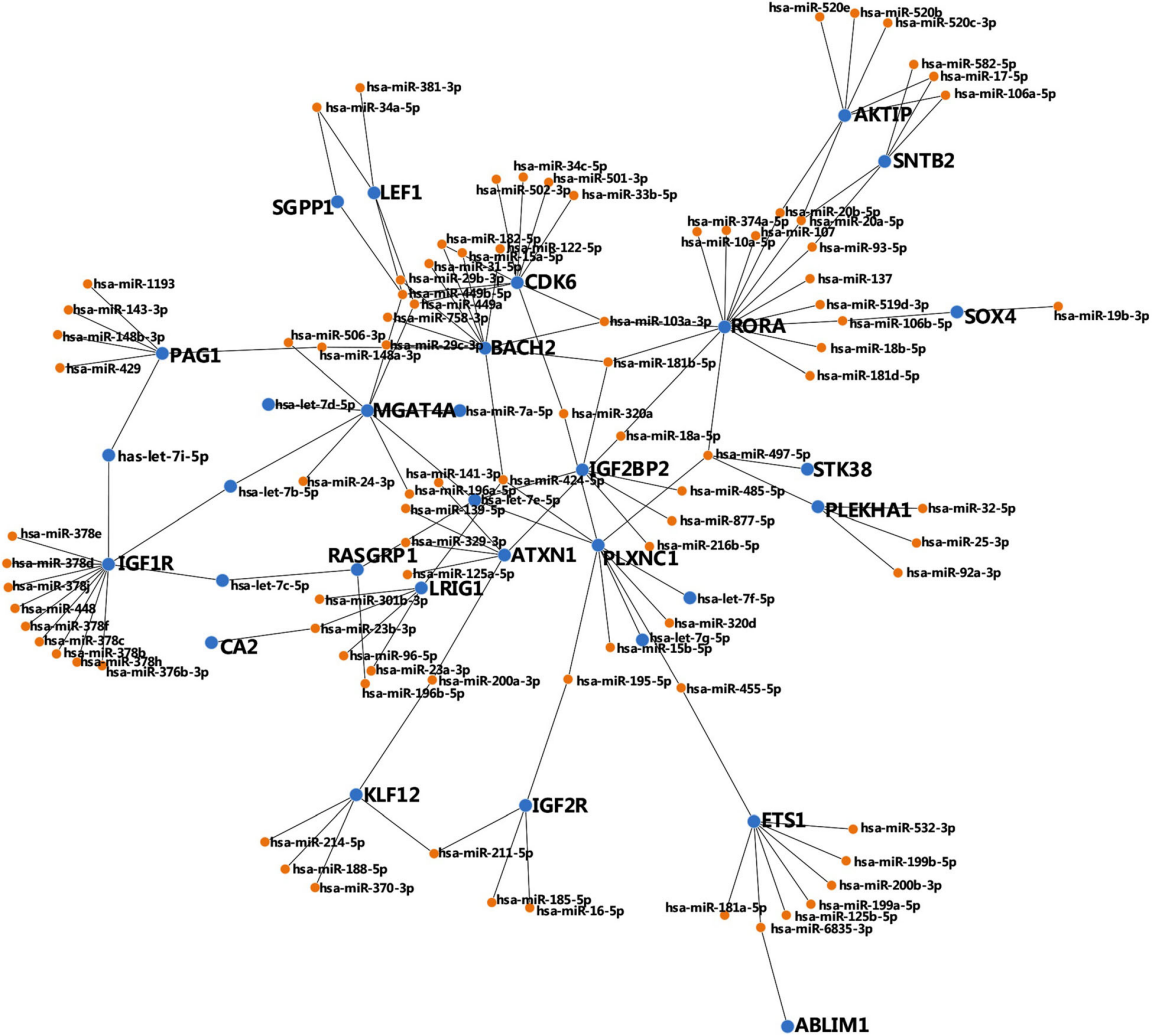
The functional miRNA-mRNA regulatory module. The module was constructed with 23 mRNAs and 97 correlated miRNAs. The functional miRNA-mRNA regulatory module are groups of genes and miRNAs with highly correlated expression patterns. It may participate in the network of cancer progression and have biological implications for AML

We used the above 139 differential expressed genes (mRNAs) to identify the correlated miRNAs which may target them. We then identified 37 mRNAs with matched 116 miRNAs (Additional file 2: Figure S2). One hundred two mRNAs were removed because these mRNAs have no matched miRNA targets. The functionally correlated miRNA-mRNA regulatory module (the center of Additional file 2: Figure S2) was then derived with 23 mRNAs and correlated 97 miRNAs (Fig. 2). Twenty-three differential expressed genes are then listed in Table 3. Among the 97 miRNAs, only the 16 miRNAs in the AML TCGA dataset were found and used for further analysis. A panel of 16 miRNA markers contained *hsa-mir-448*, *hsa-mir-320a*, *hsa-mir-378b*, *hsa-mir-378c*, *hsa-mir-378f*, *hsa-mir-378e*, *hsa-mir-378 h*, *hsa-mir-378i*, *hsa-mir-520b*, *hsa-mir-520e*, *hsa-mir-429*, *hsa-mir-137*, *hsa-mir-1193*, *hsa-mir-346*, *hsa-mir-449a*, and *hsa-mir-107*. Specifically, we named the list of differentially expressed 23 genes and 16 target-validated miRNAs the GMI signature because it integrated information from Gene expression, Mirna and miRNA-mRNA Interactions.

**Table 3:** The 23 differentially expressed genes

Gene Symbol	Mean Signal of AML Group	Mean Signal of Control Group	t statistic	Fold Change	*p*-value	q-value	Gene Feature
*SOX4*	9.112336	7.219739	7.675507	3.713032	< 1 × 10–6	< 1 × 10–6	up
*RASGRP1*	5.83589	7.652658	−7.195507	−3.522911	< 1 × 10–6	< 1 × 10–6	down
*BACH2*	4.320585	6.060818	−8.152871	−3.340891	< 1 × 10–6	< 1 × 10–6	down
*KLF12*	6.133251	7.861214	−7.870257	−3.312596	< 1 × 10–6	< 1 × 10–6	down
*LRIG1*	4.728837	6.393224	−9.836949	−3.16979	< 1 × 10–6	< 1 × 10–6	down
*ETS1*	7.934244	9.498425	−7.305467	−2.957097	< 1 × 10–6	< 1 × 10–6	down
*CA2*	7.797592	6.234079	6.061318	2.955725	< 1 × 10–6	< 1 × 10–6	up
*SGPP1*	5.2489	6.809546	−10.216815	− 2.949861	< 1 × 10–6	< 1 × 10–6	down
*AKTIP*	5.928499	7.444547	−11.312356	− 2.860065	< 1 × 10–6	< 1 × 10–6	down
*MGAT4A*	6.147385	7.656076	−6.959608	− 2.845517	< 1 × 10–6	< 1 × 10–6	down
*IGF1R*	5.953049	7.435281	−9.157838	−2.793807	< 1 × 10–6	< 1 × 10–6	down
*IGF2BP2*	8.551588	7.080512	6.331802	2.772285	< 1 × 10–6	< 1 × 10–6	up
*ATXN1*	5.977417	7.443869	−11.143182	−2.763415	< 1 × 10–6	< 1 × 10–6	down
*PLXNC1*	6.920035	8.327643	−7.413724	−2.652969	< 1 × 10–6	< 1 × 10–6	down
*PLEKHA1*	5.191966	6.576699	−6.150913	−2.611237	< 1 × 10–6	< 1 × 10–6	down
*PAG1*	6.025085	7.409445	−6.906294	−2.61056	< 1 × 10–6	< 1 × 10–6	down
*SNTB2*	3.785427	5.155895	−6.769114	−2.585543	< 1 × 10–6	< 1 × 10–6	down
*IGF2R*	6.569925	7.933466	−6.300174	−2.573159	< 1 × 10–6	< 1 × 10–6	down
*STK38*	4.463917	5.809704	−6.984927	−2.541687	< 1 × 10–6	< 1 × 10–6	down
*CDK6*	6.572063	5.241319	9.134775	2.515324	< 1 × 10–6	< 1 × 10–6	up
*LEF1*	6.517115	8.211854	−5.602032	−3.237184	< 1 × 10–6	0.000002	down
*ABLIM1*	7.040753	8.373263	−5.375175	−2.518404	0.000001	0.000004	down
*RORA*	5.041835	6.718816	−5.269869	−3.197582	0.000001	0.000006	down

Previous studies demonstrated that *Sox4* [50], *RasGRP1* [51], *RasGRP3* [51], *IGF1R* [52], *CDK6* [53], and *LEF1* [54] are the key oncogenes/tumor suppressor genes in acute myeloid leukemia. Among the GMI signature, *IGF1R* is targeted by *hsa-miR-378b*, *hsa-miR-378c*, *hsa-miR-378e*, *hsa-miR-378f*, *hsa-miR-378 h*, *hsa-miR-378i*, and *hsa-miR-448* respectively. It suggests that *hsa-miR-378* family members and *hsa-miR-448* have important regulatory functions for AML. In addition, *AKTIP* is targeted by *hsa-miR-520b* and *hsa-miR-520e*, *CDK6* is targeted by *hsa-miR-320a* and *hsa-miR-449a*, *PAG1* is targeted by *hsa-miR-429* and *hsa-miR-1193*, *RORA* is targeted by *hsa-miR-107* and *hsa-miR-137*. It confirms that *hsa-miR-520b*, *hsa-miR-520e*, *hsa-miR-320a*, *hsa-miR-449a*, *hsa-miR-429*, *hsa-miR-1193*, *hsa-miR-107*, and *hsa-miR-137* also play important roles in regulating AML. Moreover, *LEF1* is targeted by *hsa-miR-449a*, *MGAT4A* is targeted by *hsa-miR-449a*, *PLXNC1* is targeted by *hsa-miR-320a*, and *SCML4* is targeted by *hsa-miR-346*. It indicates that *hsa-miR-449a*, *hsa-miR-320a*, *and hsa-miR-346* may be very significant for the regulation of AML.

Collectively, the identified interactions between miRNAs and mRNAs suggest that the functional miRNA-mRNA regulatory module participate in the network of cancer progression and have biological implications for AML in common.

### GMI signature in survival analysis

**Fig. 3:**
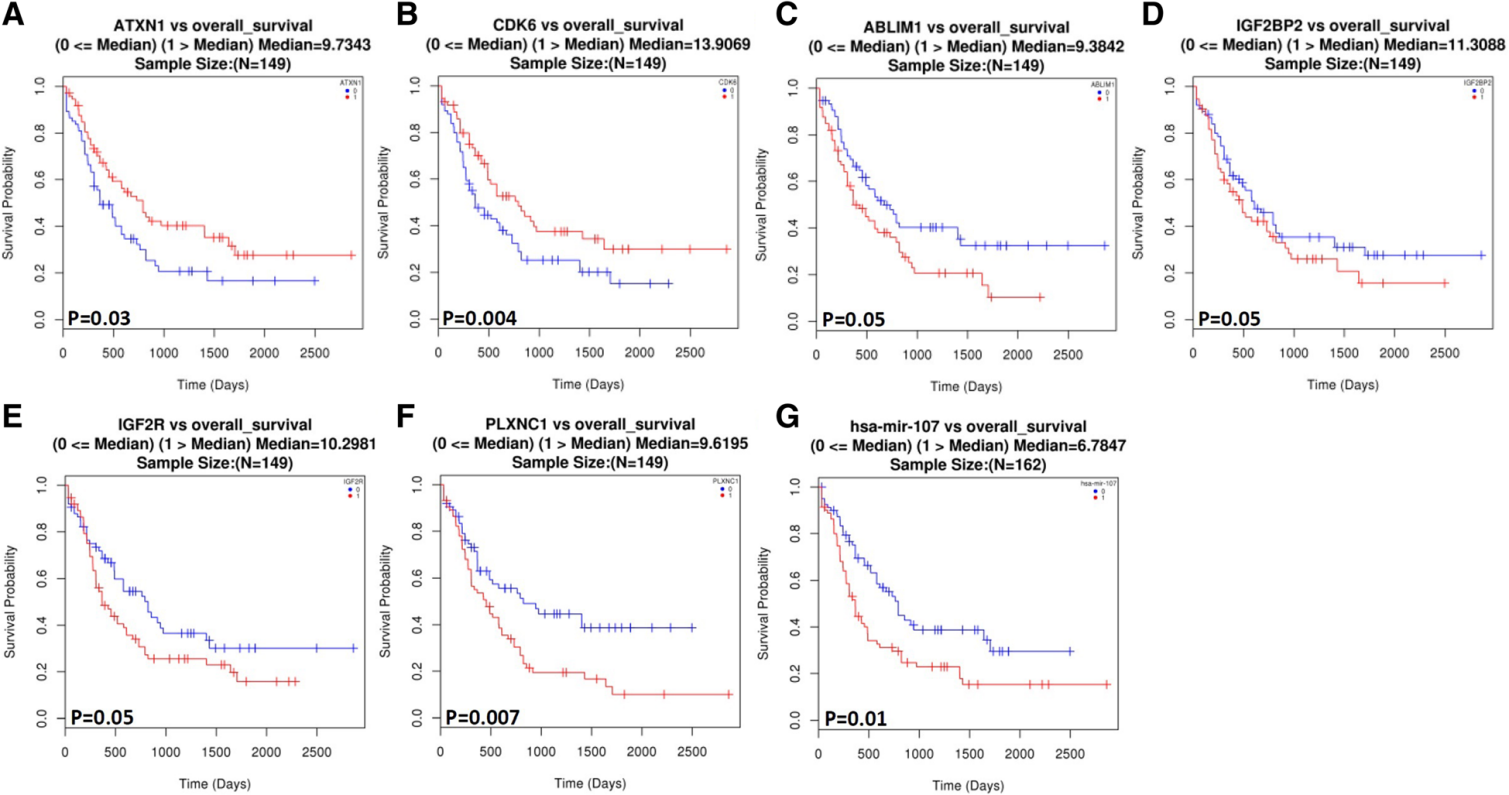
Kaplan–Meier curve analysis of seven expression signatures from GMI with LinkedOmics for the overall survival in AML patients. The median score for each gene signature is displayed in the panel label. Patients with above-median (red, label 1) and below-median (blue, label 0) scores have different overall survival rates. Horizontal axis stands for overall survival time, while vertical axis stands for overall survival probability. P values of less than 0.05 were considered to be statistically significant. A Kaplan-Meier curve of overall survival is derived for (**a**) ATXN1, (**b**) CDK6, (**c**) ABLIM1, (**d**) IGF2BP2, (**e**) IGF2R, (**f**) PLXNC1 and (**g**) hsa-mir-107 respectively

To analyze the predictive value of the GMI signature on survival we used the LinkedOmics tool. We got the survival analysis curves of each gene and target-validated miRNA from TCGA tumor samples (Fig. 3 and Additional file 2: Figure S3). Seven genes/miRNAs were found with statistical significance (*p* ≤ 0.05) including *ABLIM1*, *ATXN1*, *CDK6*, *IGF2BP2*, *IGF2R*, *PLXNC1*, and *hsa-mir-107* (Fig. 3). It is worth noting that the high-risk group has significantly worse overall survival than the low-risk group for the previous genes/miRNAs.

Similarly, we applied GEPIA for the prognosis of the GMI signature in AML from TCGA project. The patients were stratified into different prognosis subsets in a sample by determining the expression level of markers (Additional file 2: Figure S4). Survival analysis of three genes (*ABLIM1*, *p* = 0.019; *ATXN1*, *p* = 0.029; *PLEKHA1*, *p* = 0.048) revealed patient stratification with statistical significance on overall survival analysis. Interestingly, we observed that *ABLIM1* and *ATXN1* were significantly associated with overall survival for these different prognosis prediction tools.

### The GMI signature-based prognosis models improve AML survival prediction

**Fig. 4:**
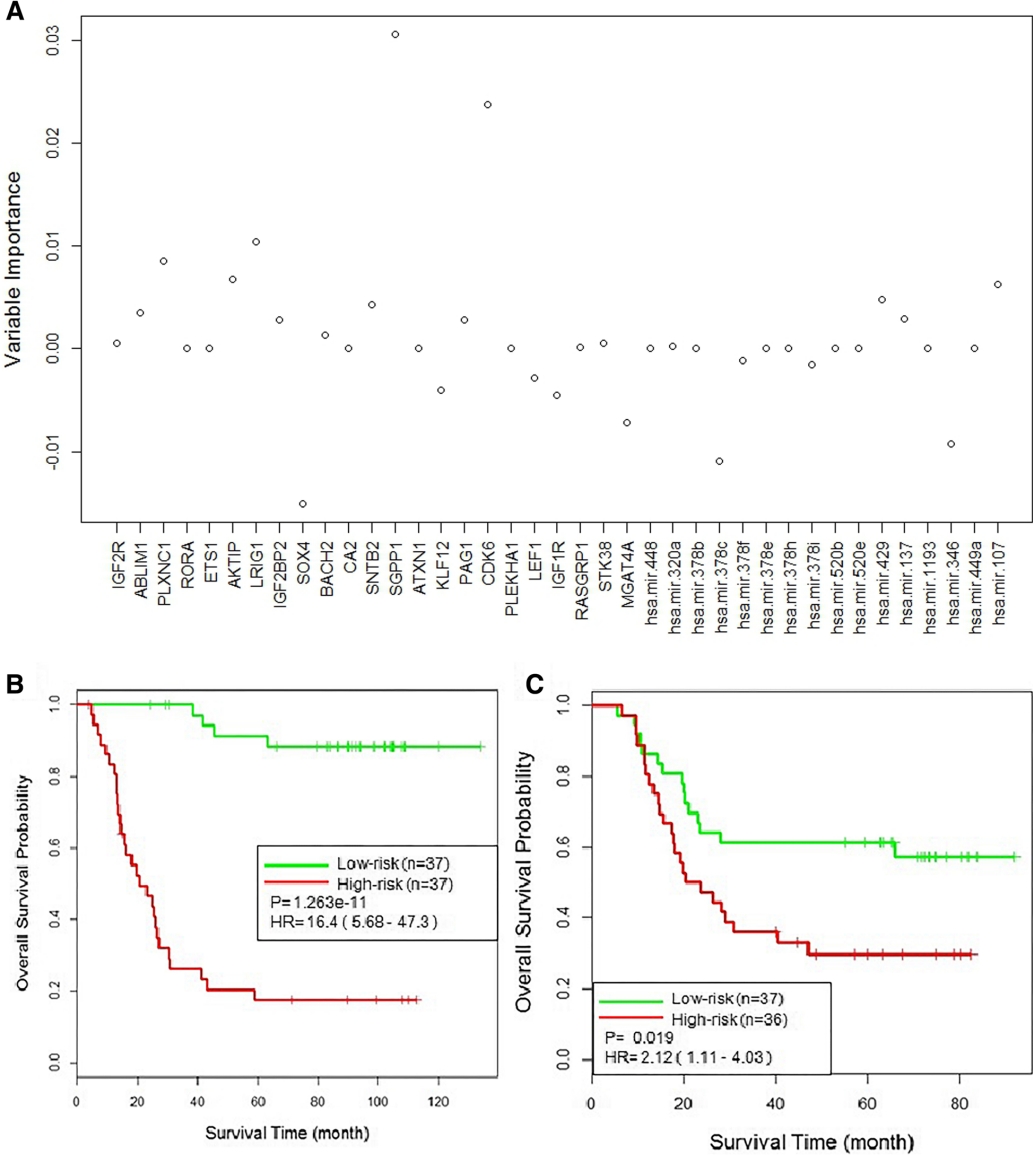
Testing the TCGAPO-derived RSF prognosis model with GMI signature on the TCGAPT. **a** Variable importance values derived from random survival forest analysis. The log-rank splitting rule was used for model development. **b** Kaplan-Meier survival curves from 5-fold cross-validation results of the TCGAPO-derived RSF prognosis model. **c** Testing the TCGAPO-derived RSF prognosis model on independent test dataset TCGAPT. Kaplan-Meier survival curves derived for the risk stratification of TCGAPT dataset

To test whether the GMI signature can predict AML recurrence, we developed prognosis model using the identified signature as features and evaluated performance of the models in independent cohorts. We developed a RSF prognosis model with the GMI signature using TCGAPO dataset. Variable importance (VIMP) is used to measure the increase (or decrease) in prediction error for the forest ensemble when a variable is randomly “noised-up” [55]. VIMP evaluates the predictive performance of the GMI signature and a large VIMP value indicates a potentially predictive variable. As shown in Fig. 4a, SGPP1 and CDK6 are potentially predictive features with larger positive VIMP values in model development.

In the RSF prognosis model development, five-fold cross validation was used to optimize the parameters for the RSF algorithm, and a full model based on the complete dataset was developed using the optimal parameters. The best performing parameters (*ntree* = 10, *mtry* = 20) were selected to build the RSF prognosis model. RSF-based scores were then calculated for individual sample. The calculated RSF-based scores displayed 78% concordance (C-index = 0.78) in the light of the real survival data in the training cohort. Based on the RSF-based scores, the samples were divided into a “high-risk” group with above-median scores and a “low-risk” group with below-median scores. As shown in Fig. 4b, the Kaplan-Meier analyses exhibited highly significant differences in time to overall survival between two different risk groups (hazard ratio [HR], 16.4; 95% confidence interval [CI], 5.68–47.3; *p =* 1.263e-11). The low-risk group had 3-year overall survival rate of 100% and the high-risk group possessed 3-year overall survival rate of 26%. The developed RSF prognosis model was applied for the independent test dataset and predictive performance was evaluated using the cohort TCGAPT. As shown in Fig. 4c, the high-risk group showed significantly worse overall survival (C-index = 0.59, hazard ratio [HR], 2.12; 95% confidence interval [CI], 1.11–4.03; *p* = 0.019) than the low-risk group. The overall survival at 3 years between low-risk and high-risk group was 63 and 35% respectively.

**Fig. 5:**
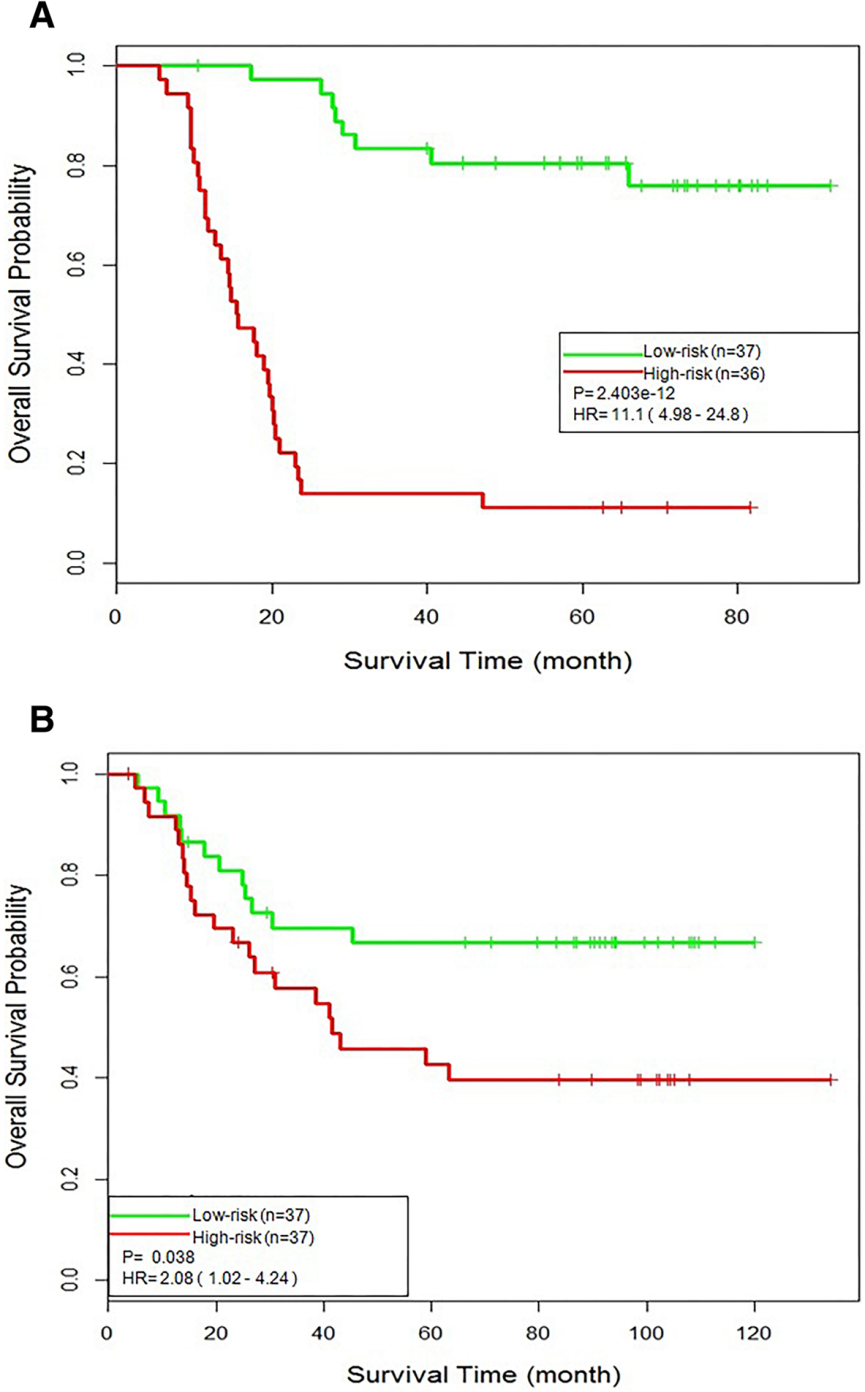
Testing the TCGAPT-derived RSF prognosis model with GMI signature on the TCGAPO. **a** Kaplan-Meier survival curves from 5-fold cross-validation results of the TCGAPT-derived RSF prognosis model. **b** Testing the TCGAPT-derived RSF prognosis model on independent test dataset TCGAPO. Kaplan-Meier survival curves derived for the risk stratification of TCGAPO dataset

To further evaluate the effectiveness of the GMI signature, we reversed the training and testing datasets by constructing RSF prognosis model based on TCGAPT dataset and testing their performance on TCGAPO dataset. Analogous results were achieved as shown in Fig. 5. As shown in Fig. 5a, scores derived from the GMI signature-based model showed 77% concordance (C-index = 0.77) when compared to the real survival data and the patients were seperated into two groups with significantly different overall survival (hazard ratio [HR], 11.1; 95% confidence interval [CI], 4.98–24.8; *p* = 2.403e-12). The 3-year overall survival rate was 81% in the low-risk group compared with 17% in the high-risk group. The TCGAPT-derived RSF prognosis model was test on independent test dataset TCGAPO. As shown in Fig. 5b, the Kaplan-Meier analyses for overall analysis illustrated the difference between the high-risk and low-risk group was highly significant (C-index = 0.58, hazard ratio [HR], 2.08; 95% confidence interval [CI], 1.02–4.24; *p* = 0.038). The overall survival at 3 years was 71% for the low-risk group in comparison with 57% for the high-risk group. These results confirmed that the GMI signature-based prognosis models could predict AML recurrence.

### Prognostic value of the GMI signature score compared to clinical variable

Samples of TCGAPT were analyzed using univariate and multivariate analyses with Cox’s proportional hazards regression to evaluate the prognostic value of the GMI signature score in combination with individual clinical variables (age, gender, race) and risk factors (WBC count, NPM mutation, FLT3-ITD mutation, CEBPA mutation, WT1 mutation). The GMI signature-based RSF model from TCGAPO was developed to test TCGAPT and achieved the GMI signature score of the samples. Univariate and multivariate Cox’s regression analyses were summarized in Table 4. The GMI signature score was significantly associated with overall survival (*p* = 0.019) in the univariate analysis and still preserved the significance (*p* = 0.02) in the multivariate analysis. As can be seen from the Table 4, there was a significant difference in overall survival with WBC count and FLT3-ITD mutation (*p* < 0.05), demonstrating the potential value of two risk factors. The GMI signature score was more highly prognostic with overall survival than individual clinical variables and risk factors when the log-rank *p* values were observed and compared. In summary, the GMI signature score represented a prognostic signature strongly associated with a higher risk of tumor recurrence.

**Table 4:** Univariate and multivariate Cox proportional hazard regression analyses of overall survival in TCGAPT. M, male; F, female; W, white; BA, Black or African American; GMI signature score was based on RSF model developed in TCGAPO

	Univariate		Multivariate	
	*p* value	HR(95% CI)	*p* value	HR(95% CI)
Age	0.25	1.03 (0.98–1.09)	0.98	1.00 (0.94–1.07)
Gender (M or F)	0.62	1.16 (0.62–2.17)	0.78	1.09 (0.58–2.06)
Race (W or BA)	0.22	1.58 (0.73–3.44)	0.21	1.69 (0.75–3.84)
WBC count	0.02	1.79 (0.91–3.95)	0.03	1.83 (0.81–4.01)
NPM mutation	0.35	1.05 (0.99–1.12)	0.41	0.98 (0.89–1.09)
FLT3-ITD mutation	0.016	2.23 (1.18–4.09)	0.03	2.08 (1.04–3.95)
CEBPA mutation	0.85	1.18 (0.82–2.37)	0.67	1.36 (0.83–3.17)
WT1 mutation	0.78	1.31 (0.86–2.35)	0.83	0.87 (0.68–1.59)
GMI score	0.019	2.11 (1.11–4.03)	0.02	2.15 (1.08–4.29)

## Discussion

MicroRNAs play crucial regulatory roles in mediating mRNA degradation with a sequence-specific manner [56]. Most previous work has focused on the experimental and computational approaches to decipher how miRNAs and genes interact in cellular network [57]. The understanding of modular organization of biological network further provides a global view on the miRNA-mRNA regulatory relationships. Previous studies have shown that the increased expression of *miR-449* causes down-regulation of oncogene *CDK6* which stimulates cell proliferation in gastric cancer [58], *miR-448* suppresses proliferation and invasion by regulating *IGF1R* in colorectal cancer cells [59], and *miR-378* family members target *IGF1R*, a key signaling molecule in rhabdomyosarcoma [60]. Based on these observations, the functional miRNA-mRNA regulatory module may be associated with cell proliferation, apoptosis and cell migration in AML. In addition, we have found that *ABLIM1* and *ATXN1* were significantly associated with overall survival for AML. In fact, loss of *CIC* or *ATXN1L* modulates sensitivity to MEK inhibition in *RAS-*mutant cancers [61]. It demonstrates that *SRGN* is crucial for regulating actin cytoskeletal organization associated with cell migration for cancer metastasis [62]. Interestingly, reduced expression of *SRGN* is accompanied by down-regulation of *ABLIM1*, *LIMA1*, *CFL1*, *RAC1*, *RAC2* and *RHOA*, concomitant with decreased cell motility [62].

The RSF prognosis models based on mRNA or miRNA expression signatures from GMI signature were developed in the cohort TCGAPO. There was a significant difference between two different risk groups with mRNA signature based RSF prognosis model (C-index = 0.72; hazard ratio [HR], 5.3; 95% confidence interval [CI], 2.15–11.8; *p =* 5.325e-3) and miRNA signature based RSF prognosis model (C-index = 0.69; hazard ratio [HR], 2.8; 95% confidence interval [CI], 1.05–6.83; *p =* 0.006). The RSF prognosis model was used for the independent test dataset TCGAPT and predictive performance was measured. No significant difference between the two different risk groups was evident for mRNA signature (C-index = 0.55, hazard ratio [HR], 1.22; 95% confidence interval [CI], 0.98–3.05; *p* = 0.05) and miRNA signature (C-index = 0.54, hazard ratio [HR], 1.69; 95% confidence interval [CI], 1.02–3.49; *p* = 0.06). These results demonstrated that RSF prognosis model based on mRNA signature or miRNA signature did not result in comparable performance as that from the GMI signature. The miRNA-mRNA interactions play an important role for achieving the predictive performance.

It has previously been observed that a 17-gene stemness score (LSC17 signature) could predict recurrence risk in AML patients [63]. The 11 genes are appeared in TCGA cohort including *CPXM1*, *EMP1*, *LAPTM4B*, *ARHGAP22*, *MMRN1*, *ZBTB46*, *AKR1C3*, *SMIM24*, *CDK6*, *NYNRIN* and *SOCS2.* The targeted 31 miRNAs (*hsa-mir-3943,hsa-mir-761,hsa-mir-765,hsa-mir-548v, hsa-mir-5739,hsa-mir-8082,hsa-mir-8089,hsa-mir-1913,hsa-mir-4290,hsa-mir-644a,hsa-mir-6132,hsa-mir-320a,hsa-mir-8054,hsa-mir-1303,hsa-mir-4313,hsa-mir-5682,hsa-mir-4426,hsa-mir-4651,hsa-mir-4447,hsa-mir-646,hsa-mir-4326,hsa-mir-922,hsa-mir-1291,hsa-mir-3911,hsa-mir-3138,hsa-mir-1179,hsa-mir-449a,hsa-mir-4481,hsa-mir-4498,hsa-mir-4657,hsa-mir-8064*) are identified with MiRWalk. The RSF prognosis models based on 11 genes and 31 miRNAs were developed in the cohort TCGAPO and independently tested in the cohort TCGAPT. There was a significant difference between two different risk groups in the training cohort (C-index = 0.79; hazard ratio [HR], 14.2; 95% confidence interval [CI], 4.97–41.7; *p =* 2.25e-8). Further test revealed that the difference between the high-risk and low-risk group was significant (C-index = 0.60, hazard ratio [HR], 2.54; 95% confidence interval [CI], 1.19–5.05; *p* = 0.03). Thus, 11 genes and 31 miRNAs could predict recurrence risk and inform patient prognosis in AML.

We developed Survival Support Vector Machine (SSVM) model based on the GMI signature for survival analysis in comparison with RSF method. Two parameters c and σ were implemented in the SSVM model, and different parameter combinations were formed for model development from each parameter among the candidate set {10^− 4^, 10^− 3^, 10^− 2^, 10^− 1^, 10^0^, 10^1^, 10^2^, 10^3^, 10^4^}. Five-fold cross validation was used to identify the optimized parameters according to the C-index value. In the TCGAPO dataset, the calculated SSVM scores showed 73% concordance (C-index = 0.73) with the real survival data. The significant differences were observed in overall survival analysis between the high-risk group and low-risk group (hazard ratio [HR], 5.39; 95% confidence interval [CI], 1.21–24.3; *P* = 0.01). When applying SSVM model on the validation dataset TCGAPT, scores showed 56% concordance (C-index = 0.56) when compared to the real survival data. The patients were separated into two different groups with overall survival (hazard ratio [HR], 1.23; 95% confidence interval [CI], 0.67–3.78; *P* = 0.06). The results indicated that RSF model achieved clearly superior performance compared to SSVM model.

For the prioritized gene expression signatures, lack of concordance is a common observation in clinical trial [64, 65]. However, several AML gene expression signatures provided the relationship with patient prognosis and survival outcome [5, 7]. It suggested that different signatures may share joint biological themes that are not obvious on the individual gene level [65]. Therefore, pathway-based analysis has been made to discover biological mechanisms underpinning concordant prognosis for different gene expression signatures [66]. Whilst it has great potential, the GMI signature based prognosis model is nevertheless limited by the small scale of currently available TCGA data. Although the performance has demonstrated that the developed RSF prognosis model is effective in improving AML survival prediction, we suppose that the concomitant increase of clinical data and large scale of training samples would ameliorate the reliability of the prognosis model for cancer treatment. Ongoing large-scale cancer genome project, e.g. TCGA project, has provided multiple molecular data for clinical cancer research. The multi-omics integration reveals the association between various genomic variables and helps to discover the complex regulatory pattern toward generated heterogeneous data including mRNA expression and miRNA expression. The NanoString technology applies color-coded molecular barcodes to hybridize directly for many different types of target molecules with high sensitivity and precision. Therefore, this emerging technology could develop the GMI signature chip as AML diagnostics tool for clinical applications.

## Conclusion

We used the pooled analysis of gene expression profiling data from 861 patients to identify differentially expressed gene expression signatures. We applied miRWalk approach to integrate multiple types of transcriptomic data and discover the functional miRNA-mRNA regulatory module. In the development of prognosis model, the GMI signature-based RSF model was used to derive the prognostic risk score and accordingly stratify the patients into a high-risk and low-risk group. The results demonstrated that the RSF prognosis model measured underlying biological characteristics which are predictive of clinical outcomes and informed the treatment in AML. In conclusion, the GMI signature based RSF prognosis model can help facilitate rational design of clinical studies by patient stratification. Notwithstanding its great potential, the GMI signature score is limited by the quality of currently available data. An important future work is to validate the clinical usefulness of the GMI signature for the developed prognosis model in AML.

## Additional files

Additional file 1: Development and validation of GMI signature based random survival forest prognosis model to predict clinical outcome in acute myeloid leukemia (XLSX 32 kb)

Additional file 2: **Figure S1.** Illustration of the volcano plot for identifying the differentially expressed genes. **Figure S2.** MiRWalk2.0 identify miRNA-mRNA interactions from an array of experimentally verified and predicted miRNA-target interaction pairs. **FigureS3.** Kaplan–Meier curve analysis of 20 expression signatures from GMI with LinkedOmics for the overall survival in AML patients. **Figure S4.** Kaplan–Meier curve analysis of GMI signature with GEPIA for the overall survival in AML patients. (DOCX 1029 kb)

## Data Availability

All data generated during this study are included in this article.

## Abbreviations

AML: Acute myeloid leukemia
FDR: False discovery rate
GEO: Gene expression omnibus
GEPIA: Gene expression profiling interactive analysis
GMI: Gene expression, mirna and miRNA-mRNA interactions
HR: Hazard ratio
OS: Overall survival
RMA: Robust multichip analysis
RSF: Random survival forest
SSVM: Survival support vector machine
TCGA: The cancer genome atlas
VIMP: Variable importance
