# Supplementary material for: Development and validation of GMI signature based random survival forest prognosis model to predict clinical outcome in acute myeloid leukemia
Source: BMC Med Genomics. 2019 Jun 26;12:90. doi: 10.1186/s12920-019-0540-5 (PMC6595612; doi:10.1186/s12920-019-0540-5)
Supplement: Supplementary file 2 — Figure S1. Illustration of the volcano plot for identifying the differentially expressed genes. Figure S2. MiRWalk2.0 identify miRNA-mRNA interactions from an array of experimentally verified and predicted miRNA-target interaction pairs. FigureS3. Kaplan–Meier curve analysis of 20 expression signatures from GMI with LinkedOmics for the overall survival in AML patients. Figure S4. Kaplan–Meier curve analysis of GMI signature with GEPIA for the overall survival in AML patients. (DOCX 1029 kb) [file 12920_2019_540_MOESM2_ESM.docx]

**Additional file 2**

**Development and validation of GMI signature based random survival forest prognosis model to predict clinical outcome in acute myeloid leukemia**

**Mingguang Shi*, Guofu Xu**

**Figure S1.** Illustration of the volcano plot for identifying the differentially expressed genes.

**Figure S2.** MiRWalk2.0 identify miRNA-mRNA interactions from an array of experimentally verified and predicted miRNA-target interaction pairs.

**FigureS3.** Kaplan–Meier curve analysis of 20 expression signatures from GMI with LinkedOmics for the overall survival in AML patients.

**Figure S4.** Kaplan–Meier curve analysis of GMI signature with GEPIA for the overall survival in AML patients.


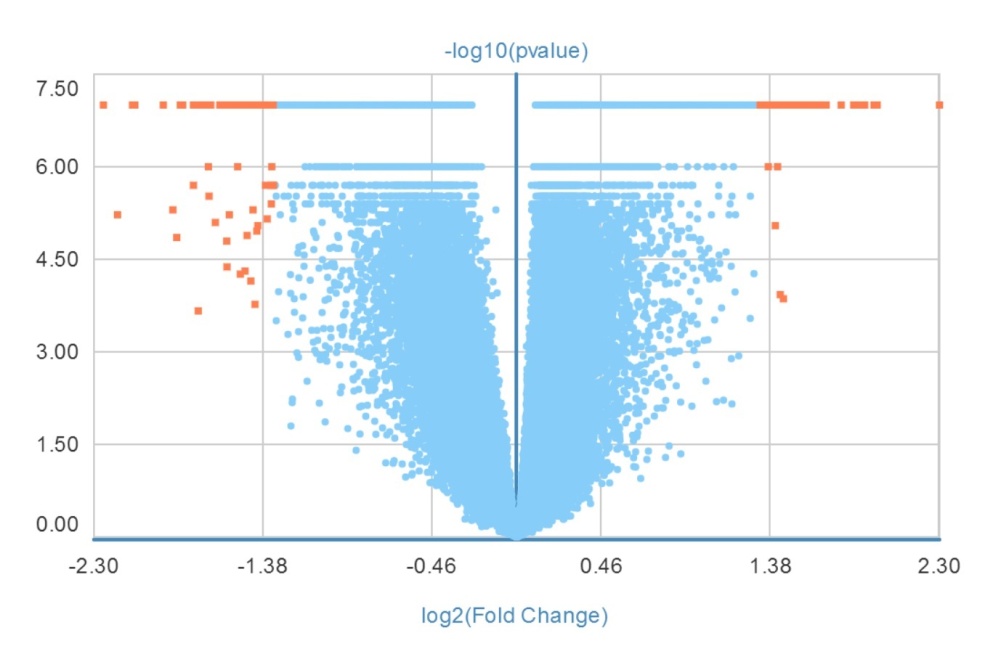


**Figure S1.** Illustration of the volcano plot for identifying the differentially expressed genes.


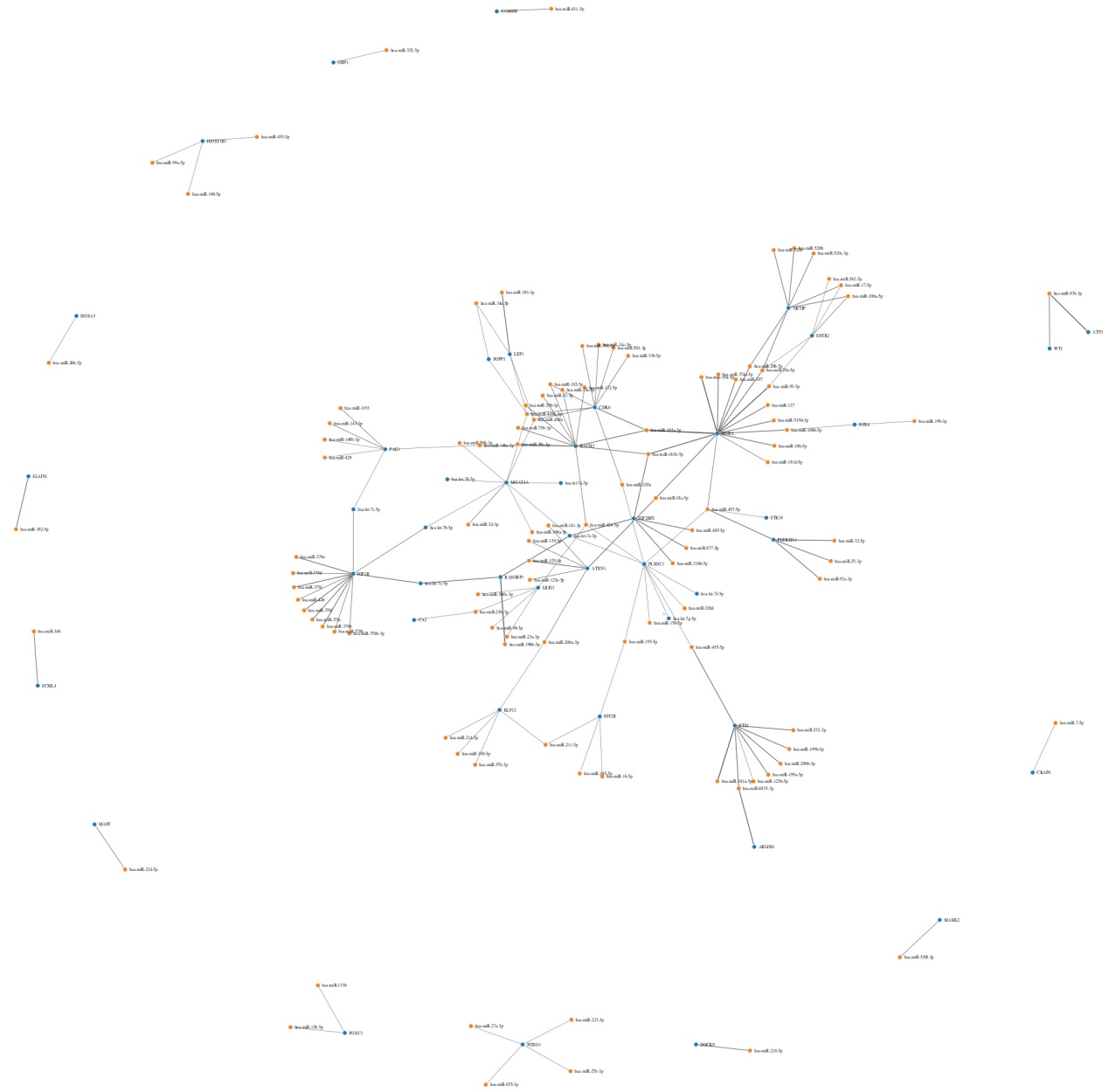


**Figure S2.** MiRWalk2.0 identify miRNA-mRNA interactions from an array of experimentally verified and predicted miRNA-target interaction pairs. 37 mRNAs were recognized with matched 116 miRNA targets.


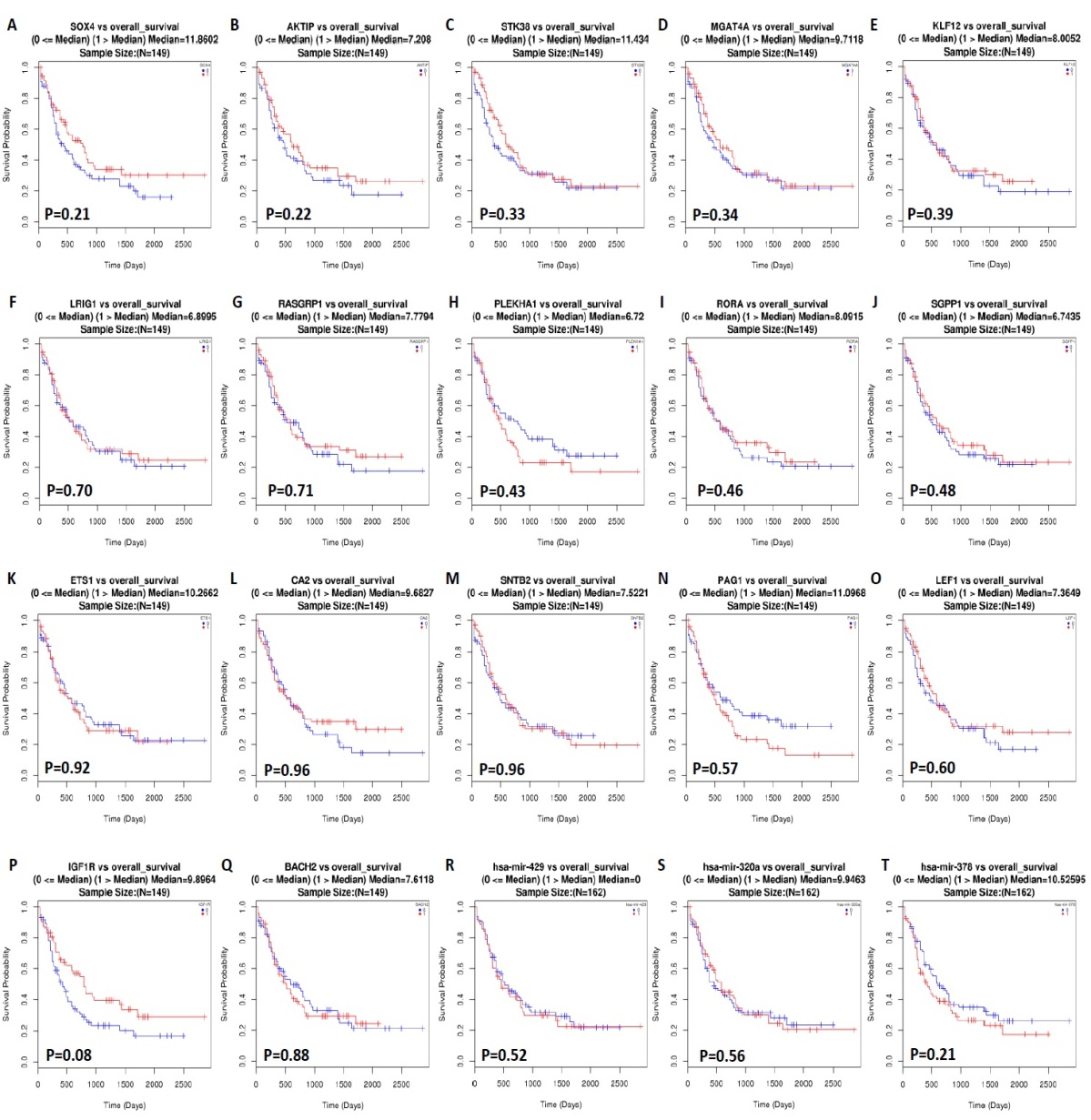


**Figure S3.** Kaplan–Meier curve analysis of 20 expression signatures from GMI with LinkedOmics for the overall survival in AML patients. Patients with above-median (red, label 1) and below-median (green, label 0) scores have different overall survival rates. Horizontal axis stands for overall survival time, while vertical axis stands for overall survival probability.


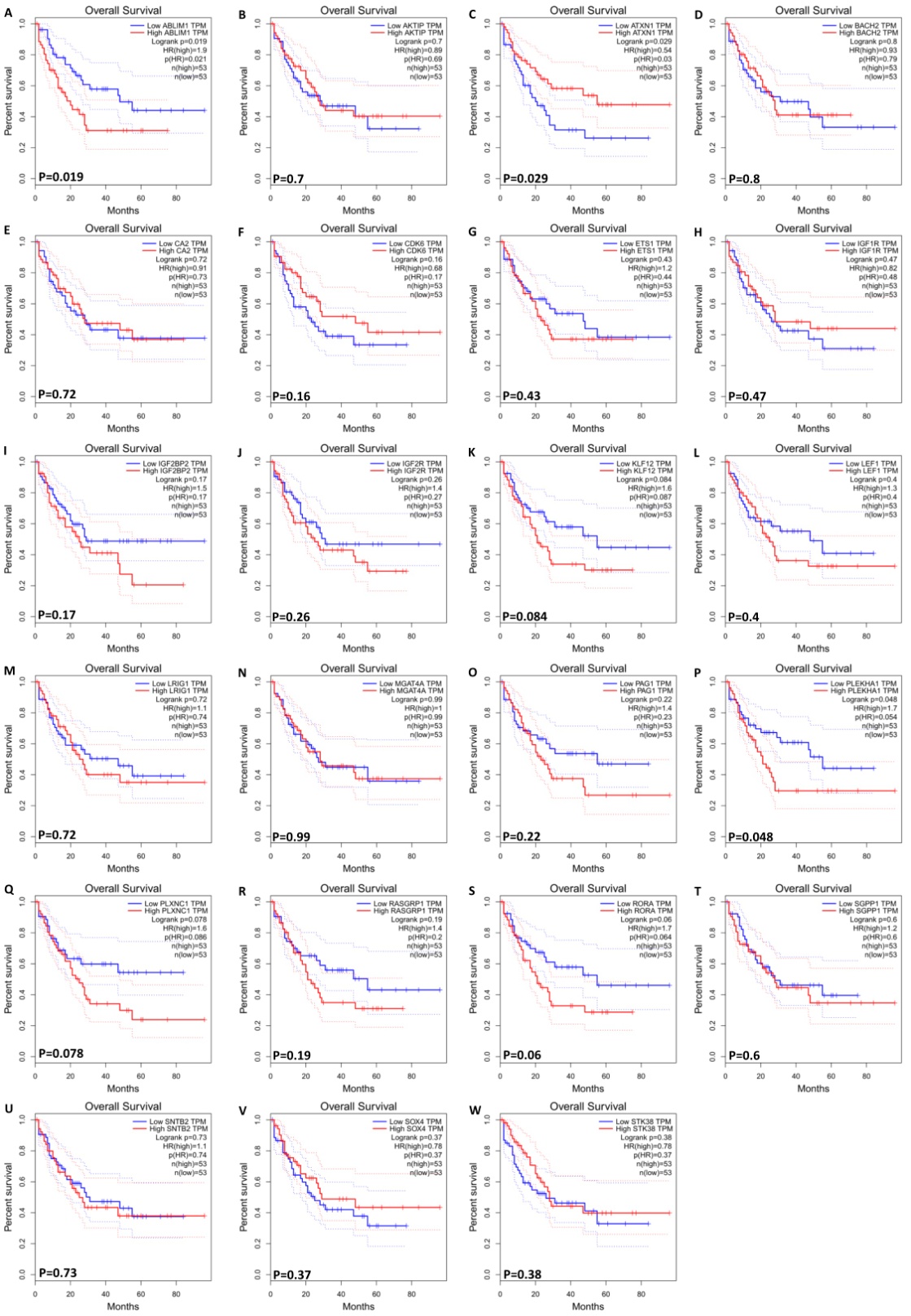


**Figure S4.** Kaplan–Meier curve analysis of GMI signature with GEPIA for the overall survival in AML patients. Patients with above- (red) and below- (blue) median mRNA abundance had different survival rates in mRNA expression from TCGA project. Horizontal axis stands for overall survival time, while vertical axis stands for overall survival probability.
